# Paraneoplastic Teratoma-associated Anti-N-Methyl-D-Aspartate Receptor Encephalitis: The First Published Report from Saudi Arabia

**DOI:** 10.7759/cureus.3527

**Published:** 2018-10-31

**Authors:** Mohammed Abuzaid, Osama Alomar, Hany Salem

**Affiliations:** 1 Department of Obstetrics and Gynecology, King Faisal Specialist Hospital and Research Centre, Riyadh, SAU

**Keywords:** saudi arabia, paraneoplastic, teratoma, ovary, nmda receptor, encephalitis, oophorectomy, case report

## Abstract

Paraneoplastic teratoma-associated anti-N-methyl-D-aspartate (anti-NMDA) receptor encephalitis is a lately introduced disease that was first documented in 2007. In a recent systemic review in 2014, only a total of 174 cases of teratoma-associated anti-NMDA receptor encephalitis was reported. Herein, to the best of our knowledge, in Saudi Arabia, we report the first ever case of mature ovarian teratoma-associated anti-NMDA receptor encephalitis in a 21-year-old Saudi woman who presented to clinical attention with a nine-day history of neuropsychiatric symptoms preceded by a two-day flu-like illness. Central nervous system (CNS) examination was remarkable for confusion and an inability to move her lower limbs. Abdominal examination was remarkable for mild right lower quadrant tenderness without palpable organomegaly. Initial laboratory findings were remarkable for high CA-125 level of 205 units/ml (normal: 0 - 35 units/ml) and CA 19-9 level of 121 units/ml (normal: 0 - 37 units/ml). Cerebrospinal fluid (CSF) examination showed lymphocytic pleocytosis and oligoclonal bands. Computed tomography (CT) scan of the abdomen and pelvis showed a 7.2 x 6.3 x 5.5 cm mass of the right ovary that was highly suspicious for a mature teratoma with fat densities and calcified foci. Serum and CSF tested positive for anti-NMDA receptor antibodies. The patient underwent right oophorectomy and the final histopathological diagnosis was confirmed. Postoperatively, the patient had an uneventful postoperative course and did not receive adjuvant secondary immunotherapies. One day following the surgery, her neuropsychiatric symptoms improved dramatically. At a six-month follow-up at the outpatient clinic, the patient was symptom-free

## Introduction

Paraneoplastic teratoma-associated anti-N-methyl-D-aspartate (anti-NMDA) receptor encephalitis is a lately introduced disease that was first documented in 2007 [1]. In a recent systemic review by Acien and colleagues in 2014, only a total of 174 cases of teratoma-associated anti-NMDA receptor encephalitis was reported [2]. Herein, to the best of our knowledge, in Saudi Arabia, we report the first ever case of ovarian teratoma-associated anti-NMDA receptor encephalitis in a 21-year-old Saudi woman who presented to clinical attention with a nine-day history of neuropsychiatric symptoms preceded by a two-day flu-like illness.

## Case presentation

A 21-year-old Saudi female, previously healthy, presented to the emergency department with a nine-day history of hallucinations, delusions, insomnia, cognitive decline, recurrent episodes of loss of body tone, and inability to walk. These symptoms were preceded by a two-day history of a non-specific headache and prodromal flu-like illness. The patient reported similar symptoms six weeks previously, followed by severe respiratory distress requiring admission to an intensive care unit (ICU) for intubation and mechanical ventilation. Her past surgical and family history were unremarkable.

On general physical examination, her vital signs were unremarkable and the patient was drowsy and lethargic. Central nervous examination (CNS) was remarkable for confusion, disorientation (to person, place, and time), delayed responses, speaking in small sentences, inability to move her lower limbs, and being uncooperative. Abdominal examination was remarkable for mild right lower quadrant tenderness without organomegaly or palpable masses.

Initial laboratory findings were remarkable for an elevated serum level CA-125 of 205 units/ml (normal: 0 - 35 units/ml) and CA 19-9 of 121 units/ml (normal: 0 - 37 units/ml).

**Figure 1:**
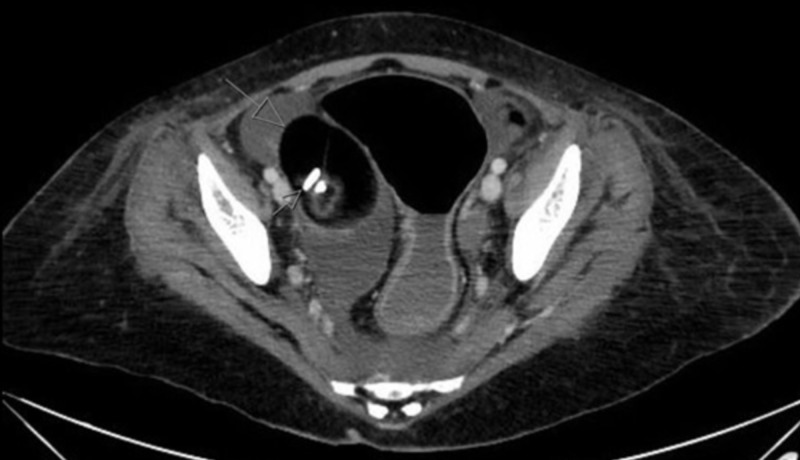
Preoperative CT scan of the abdomen/pelvis Cross-sectional computed tomography (CT) scan of abdomen/pelvis showing a 7.2 x 6.3 x 5.5 cm mass of the right ovary that was highly suspicious for a mature teratoma with fat densities and calcified foci

Magnetic resonance imaging (MRI) of the brain showed no evidence of acute of intracranial abnormality. An electroencephalogram (EEG) was unremarkable. Cerebrospinal fluid (CSF) examination showed lymphocytic pleocytosis and oligoclonal bands. Electromyogram (EMG) and nerve conduction study (NCS) of the lower limbs showed mild non-specific myopathic changes. Computed tomography (CT) scan of the abdomen and pelvis showed a 7.2 x 6.3 x 5.5 cm mass of the right ovary that was highly suspicious for a mature teratoma with fat densities and calcified foci (Figure 1).

Psychiatric consultation was recommended for the purpose of (a) pulse methylprednisolone therapy to treat the psychotic symptoms of delusions and hallucinations and (b) melatonin therapy to aid in sleep. However, the patient showed no improvement.

In view of a possible paraneoplastic teratoma-associated anti-NMDA receptor encephalitis, samples were sent to the Mayo Clinic Hospital, Rochester, Minnesota, USA to test for the presence of anti-NMDA receptor antibodies. The results came back positive for anti-NMDA receptor antibodies in the serum and CSF. Subsequently, the patient was started on a five-day course of intravenous methylprednisolone, 1 gm per day, and intravenous immunoglobulins (IVIG), 400 mg/kg/day. The neuropsychiatric symptoms showed some improvement. Afterward, the patient was subjected to a right oophorectomy.

**Figure 2:**
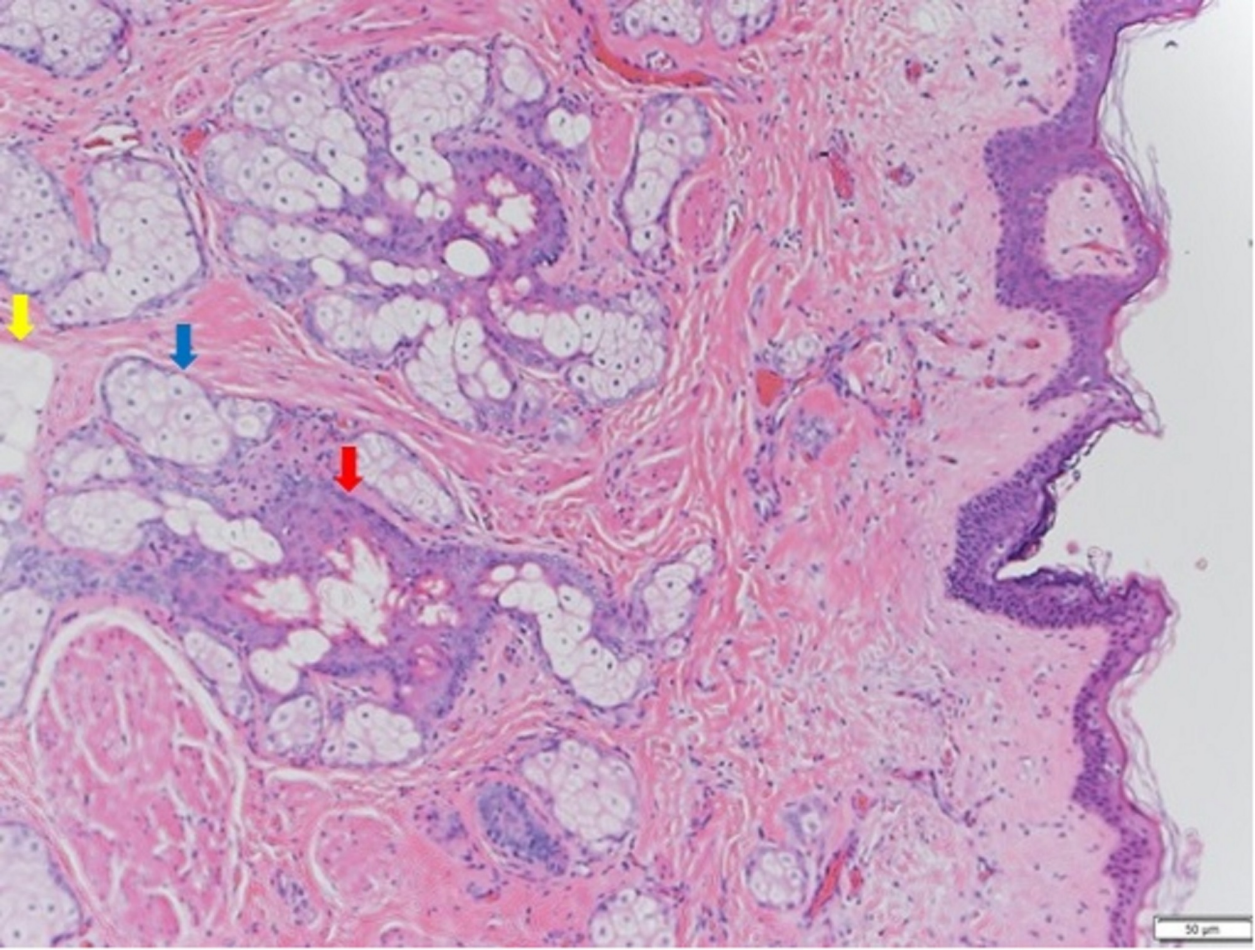
Hematoxylin and eosin (H&E) stain The ovarian cyst was lined by ectodermal derivatives containing skin adnexa, such as hair follicles (red arrow) and sebaceous glands (blue arrow). Fat cells were also identified (yellow arrow).

Macroscopically, the resected mass had multi-loculated cystic cavities filled with fatty, sebaceous, tooth, and hair elements. Microscopically, the ovarian cyst was lined by ectodermal derivatives containing skin adnexa, such as hair follicles, arrector pili muscles, and sebaceous glands, as well as the identification of fat cells (Figure 2). The final histopathological diagnosis was mature teratoma associated with paraneoplastic anti-NMDA receptor encephalitis.

Postoperatively, the patient had an uneventful postoperative course. One day following the right oophorectomy procedure, her neuropsychiatric symptoms improved dramatically. The patient did not receive any secondary immunotherapies. At a six-month follow-up at the outpatient clinic, the patient was symptom-free.

## Discussion

To a larger degree, our patient had a classical "clinical" presentation of anti-NMDA receptor encephalitis. The clinical suspicion of anti-NMDA receptor encephalitis diagnosis was further supported by an imaging finding of an underlying ovarian mass. Clinically, the most typical presentation of anti-NMDA receptor encephalitis often encompasses a combination of neurologic, psychiatric, and autonomic symptoms. This combination of symptoms is frequently accompanied by a viral prodrome, such as fever, headache, nausea, vomiting, diarrhea, and flu-like illness [3]. Within a few days to two weeks, rapidly progressive neuropsychiatric symptoms, such as psychosis, hallucinations, convulsions, behavioral disturbances, insomnia, mania, paranoia, and short-term memory deficits, emerge as the initial symptoms; therefore, psychiatric consultation is often obtained [3]. Later, autonomic symptoms, such as ataxia, dyskinesia, hypotension, hypoventilation, and coma, may progress and require supportive attention in the intensive care unit (ICU) [3].

The vast majority of anti-NMDA receptor encephalitis cases take place in childbearing female patients (80%) [4]. The median age at clinical presentation is roughly 21 years old [3]. This gender and age predilection matched our patient's in the presented study. 

Our patient had a mature teratoma (with fat and calcified foci) that was detected on a computed tomography (CT) scan of the abdomen and pelvis during her admission workup. In the largest published retrospective case series of 400 patients, almost all cases of anti-NMDA receptor encephalitis were associated with underlying ovarian teratomas (98%) [3]. Additionally, the histopathological type of the ovarian teratoma was mostly a mature teratoma (60%) but also could include an immature teratoma or combined mature/immature teratoma [2]. The teratoma may be identified soon after admission through the utilization of CT and MRI modalities. However, sometimes the underlying teratoma may be radiologically invisible and hence not identified until a couple of days or years later following the clinical presentation of anti-NMDA receptor encephalitis [4]. Also, it may only be detected histopathologically following an oophorectomy [2].

Due to the under-recognized knowledge of anti-NMDA receptor encephalitis, the diagnosis can be easily missed by inexperienced physicians [5]. In fact, the diagnosis is more likely to be missed by physicians who belong to departments other than medicine and neurological sciences. However, in our case, the patient was treated in a highly specialized tertiary healthcare center with competent neurologists and gynecologic oncologists.

The differential diagnosis in our case included autoimmune (e.g., systemic lupus erythematosus), metabolic (e.g., Wernicke’s encephalopathy), infectious (e.g., herpes simplex virus encephalitis), and neoplastic (e.g., brain lymphoma) etiologies [5]. Herpes simplex viral (HSV) encephalitis has been established as a potential trigger for the development of latent autoimmune encephalitis. Specifically, several studies showed that around 20 - 30% of patients who are negative for the NMDA receptor antibody (at time of HSV encephalitis) will seroconvert to a positive NMDA receptor antibody during a relapse of symptoms. Additionally, a small number of patients may potentially develop antibodies to the NMDA receptor, even in the absence of clinical symptoms [6-8]. Generally, the above-mentioned differential diagnoses can be excluded based on physical examination, laboratory results, and imaging findings [8]. In our case, the clinical presentation of psychosis (preceded by two days of flu-like prodrome), the identification of an underlying ovarian mass on imaging, and the highly specific serological tests narrowed down the diagnosis with high certainty.

A definitive diagnosis of anti-NMDA receptor encephalitis is established when immunoglobulin G (IgG) antibodies targeted against the GluN1 (also known as NR1) subunit of the NMDA receptor are identified in the serum or CSF [9]. CSF antibodies seem to be largely more correlated with clinical outcome than serum antibodies [10]. Serum IgG is often associated with false-positive and false-negative results; on the other hand, CSF IgG is highly sensitive and specific for anti-NMDA receptor encephalitis. Thus, CSF IgG antibodies must be tested (with or without serum antibodies) in diagnosing anti-NMDA receptor encephalitis [11]. Immunoglobulin A (IgA) and immunoglobulin M (IgM) antibodies are often elevated in patients with various chronic neurological conditions (i.e., schizophrenia). Therefore, they are not specific against the NMDA receptor, and they do not induce changes to NMDA receptors in *in-vivo* animal studies. Thus, IgA and IgM are not clinically useful in the diagnosis of NMDA receptor encephalitis [12-13]. Our hospital did not have the facility for this serum/CSF serological test, and hence patient's samples were sent externally to an international collaborative healthcare institute.

In patients with anti-NMDA receptor encephalitis, the MRI may be abnormal in only 33% of patients, while EEG irregularities are often observed in more than 90% of patients [9]. Relevant to the presented case, our patient exhibited unremarkable MRI and EEG findings.

Overall, anti-NMDA receptor encephalitis is roughly associated with a 4 - 7% fatality [2, 14]. Despite the hazard of mortality, approximately 80% of patients managed with first-line immunotherapy and early surgical tumor resection exhibit favorable outcomes, in terms of a faster therapeutic response, an improved neurological aftermath, a reduced likelihood of relapse, and a decreased probability of needing a second-line immunotherapy [3, 14]. Options of first-line immunotherapy commonly include plasmapheresis, IVIG, or steroids, whereas options of second-line immunotherapy (postoperatively) commonly include rituximab, cyclophosphamide, or both [3]. Our patient was treated successfully with intravenous methylprednisolone, IVIG, and surgical excision of the underlying paraneoplastic trigger. 

Prognosis of anti-NMDA receptor encephalitis is not poor. In March 2017, Zhang and colleagues published a systematic review of all reported cases of anti-NMDA receptor encephalitis (n = 432) [15]. Outcomes of anti-NMDA receptor encephalitis were classified according to the modified Rankin Scale (mRS) score for degree of disability and reported to be “full recovery (score: 0 - 1)”, “substantial improvement (score: 2 - 4),” and “limited improvement/death (score: 5 - 6)” in 44%, 47%, and 9% of all patients, respectively. Relapse of anti-NMDA receptor encephalitis is not uncommon, and it occurs in around 15 - 24% of patients [14]. Thus, long-term follow-up is greatly advised. Disease relapse has a high likelihood to occur in patients who did not receive immunotherapy with the initial presentation [14].

## Conclusions

In conclusion, herein, we report the first case of paraneoplastic teratoma-associated, anti-NMDA receptor encephalitis in Saudi Arabia. Although rare, it should be considered in the differential diagnosis of women of childbearing age presenting with unexplained neuropsychiatric symptoms. Also, imaging should be undertaken to search for an underlying paraneoplastic ovarian mass.
